# The economic burden of TB-affected households in DR Congo

**DOI:** 10.5588/ijtld.21.0182

**Published:** 2021-11-01

**Authors:** M. Kaswa, G. Minga, N. Nkiere, B. Mingiedi, G. Eloko, P. Nguhiu, I. Garcia Baena

**Affiliations:** 1 Programme National de Lutte contre la Tuberculose, Ministére de la Santé Publique, Kinshasa, DR Congo; 2 Service de Microbiologie, Département de Biologie médicale, Université de Kinshasa, Kinshasa, DR Congo; 3 World Health Organization, DR Congo Office, Kinshasa, DR Congo; 4 Institut National de Statistique, Ministère du Plan, Kinshasa, DR Congo; 5 Programme National des Comptes Nationaux de la Santé, Ministère de la Santé Publique, Kinshasa, DR Congo; 6 Health Economics Research Unit, Kenya Medical Research Institute - Wellcome Trust Research Programme, Nairobi, Kenya; 7 Global TB Programme, World Health Organization, Geneva, Switzerland

**Keywords:** tuberculosis, catastrophic cost, patient costs, out-of-pocket expenditures, survey, DR Congo

## Abstract

**BACKGROUND:**

The Democratic Republic of Congo’s free TB care policy and recent progress with universal health coverage are insufficient to remove barriers to TB care access and adherence. As there were no nationally representative data on the economic burden borne by TB patients, the TB programme conducted a national survey to assess the proportion of TB patients facing catastrophic costs, which could also serve as a baseline for monitoring progress.

**METHODS:**

A national survey with retrospective data collection and projection, following WHO methods, was administered to 1,118 patients in 43 treatment zones. Each patient was interviewed once on costs, time loss, coping measures, income, household expenditure and asset ownership. Total costs were expressed as a percentage of annual household expenditure.

**RESULTS:**

In 2019, 56.5% of households affected by TB experienced costs above 20% of their annual household expenditure. Mean costs amounted to respectively US$400 (range: 328–471) and US$1,224 (range: 762–1,686) per episode of first-line and drug-resistant TB. The risk of catastrophic costs increased with hospitalisation, drug resistance status and lower economic status. Half of households resorted to coping strategies and experienced food insecurity. Only 7.5% received social support.

**CONCLUSION:**

TB-affected households incur on average a cost of US$549, despite free TB care policy. Mitigating this burden with medical cost reductions, social and labour market measures will be key.

## Abstract (FR)

**CONTEXTE:**

La politique de gratuité des soins antituberculeux de la République démocratique du Congo et les récents progrès en matière de couverture universelle des maladies ne suffisent pas à éliminer les obstacles liés à l’accès aux soins antituberculeux et à l’observance. N’ayant pas données représentatives au niveau national sur le fardeau économique supporté par les patients TB, le programme de lutte contre la TB a réalisé une enquête nationale pour évaluer la proportion de patients TB confrontés à des coûts catastrophiques, qui pourrait également servir de référence pour le suivi des progrés.

**MÉTHODES:**

Une enquête nationale avec recueil de données rétrospectives et réalisation de projections, conformément aux méthodes de l’OMS, a été réalisée auprès de 1 118 patients issus de 43 zones de traitement. Chaque patient a été interrogé une fois sur les points suivants : coûts, perte de temps, mesures prises face aux difficultés, revenus, dépenses du foyer et propriété d’actifs. Les coûts totaux ont été exprimés sous forme de pourcentage des dépenses annuelles des foyers.

**RÉSULTATS:**

En 2019, 56,5% des foyers touchés par la TB ont subi des coûts supérieurs à 20% de leurs dépenses annuelles. Les coûts moyens s’élevaient respectivement à 400 dollars américains (écart : 328-471) et 1 224 dollars américains (écart : 762-1 686) par épisode de TB sous traitement de première intention et de TB pharmacorésistante. Le risque de coûts catastrophiques était accru en cas d’hospitalisation, de résistance thérapeutique et de statut économique moins élevé. La moitié des foyers ont dû adopter des stratégies pour faire face aux difficultés et ont connu une insécurité alimentaire. Seuls 7,5% ont bénéficié d’une aide sociale.

**CONCLUSION:**

Les foyers touchés par la TB ont des coûts s’élevant en moyenne à 549 dollars américains, malgré la politique de gratuité des soins antituberculeux. Il sera donc essentiel d’atténuer ce fardeau en réduisant les coûts médicaux et en prenant des mesures sociales et en faveur de l’emploi.

Assessing the economic burden borne of patients affected by TB and establishing progress towards zero suffering from catastrophic costs target are key to monitoring progress towards global and the Democratic Republic of Congo’s strategy to end TB.^1^ Survey evidence in the African region to date has provided valuable entry points to multi-sectoral action plans to combat TB,^2^ including better linkages to social protection systems in line with the WHO’s End TB Strategy and universal health coverage (UHC) targets.^3^ Access to quality health-care with financial protection at the heart of UHC are critical to building resilient health systems capable of bearing epidemic shocks when paired with poverty alleviating measures (Sustainable Development Goal [SDG] 1) and stronger governance structures (SDG 16).^4^

DR Congo (population: 87 million; 72% living on less than US$1.90 a day^5^) has a fragile health system, rebuilt following years of conflict (2002) dealing with health emergencies (e.g., Ebola, COVID) while combating TB. TB affects 1 in 300 Congolese,^*^ kills 1 in 2000 infected, and was the third cause of mortality in 2019.^6^ In 2018, 4.5% of the gross domestic product (GDP) was invested in health. TB patients benefit from free TB services upon notification, with 2,050 centres delivering TB care for 180,609 patients in 2019.^7^,^8^ In 2019, DR Congo spent US$18.5 per capita, of which US$7.7 was financed out of pocket.^7^ The WHO estimates that delivering TB care in 2019 cost respectively US$150 and US$4,694 for patients on first-line (i.e., drug-susceptible TB [DS-TB]) and drug-resistant TB (DR-TB) treatment.^2^ However, costs supported by TB-affected households were unknown to date. With only 41% of patients accessing quality essential services, many households forego seeking care in the formal health sector.^9^,^10^

This is the first national representative survey evaluating the economic burden faced by TB-affected patients; our objectives were to establish a baseline to measure progress towards the zero catastrophic cost target of global and national TB strategy, as well as to document the magnitude and main determinants of the costs incurred by TB patients’ households and most affected population strata.

## METHODS

This was a national representative, cluster random-sampled, cross-sectional, facility-based survey, with retrospective data collection and with extrapolation of costs within the treatment period in line with WHO recommendations.^11^

### Sampling

The sampling frame included 518 health facilities in the TB programme network. Assuming a 30% proportion of catastrophic costs, a design effect of 2.0 and a precision level of 5%, the sample size was calculated as 1,118; 43 health zones (clusters) were randomly sampled, and facilities selected with probability proportional to cases notified in 2017. Per facility, 26 patients were then consecutively sampled. All adults and children on treatment for TB for at least 14 days either in intensive or continuation phase from July to October 2019 were eligible, following WHO design.^11^

### Data collection

From July to October 2019, 31 trained interviewers administered paper or electronic questionnaires, adapted from the WHO generic instrument,^11^ in French or the local languages,^12^ to up to 26 patients per facility on random days. Each consenting adult or child’s caregiver was interviewed once to obtain their medical and non-medical costs incurred, time loss, health care utilisation history, household coping strategies and perceived social impact. Patients self-reported individual and household income (predisease, at diagnosis and during treatment), annual household expenditure and household assets per pretested questionnaires from previous national surveys,^13^ and the WHO survey manual definitions.^11^ Only respondents interviewed during the intensive phase would be questioned on related pre-diagnostic costs and time loss. Retrospective costs, income and spending were collected in Congolese francs (CDF) and converted using US$1 = CDF1,653.^14^

### Analysis

#### Costs borne by TB-affected patients

Direct medical (visits, drugs and hospitalisation), non-medical (i.e., transport, nutritional supplements, food and accommodation) and indirect costs (i.e., loss of income) were estimated per TB-affected household. Following WHO methods,^11^ retrospective costs and time loss reported by each patient were extrapolated mechanistically within the phase, beyond and until planned treatment completion using median values reported by survey patients. This allowed capturing of data on diagnosis and treatment costs borne by patients per TB or DR-TB episode, defined as the time from self-reported onset of TB-related symptoms until end of treatment or death. Total costs summed the direct medical and non-medical, as well as indirect costs measured as a valuation of time loss. Episode costs and cost drivers were evaluated across household expenditure quintiles. Total costs were expressed as a percentage of annual household expenditure.

#### Income and poverty levels

Annual household expenditure was used as the primary method for determining household ability to pay. Collected self-reported income pre- and post-disease were not used as the main measure for household ability to pay nor as indirect cost measure; instead, annual household expenditure and time loss valued at a fixed hourly wage rate were used. Income loss was estimated using the human capital and equality of wages method, whereby hours lost in care were multiplied by minimum wage of CDF814 per hour.^15^ We report estimated annual household expenditure distribution of study population by expenditure quintile and resistance status. We evaluated pre-disease household poverty levels by comparing unadjusted self-reported income from respondents against the international poverty threshold of US$1.90 purchasing power parity (converted to CDF in 2011, then inflated using GDP deflator^16^ 2011 vs. 2019).

#### Catastrophic costs

The patient’s costs were compared against their household’s annual expenditure. Each household was given a binary value representing whether or not they incurred catastrophic (>20% of annual household expenditure) total costs due to their TB disease. Sensitivity analysis was conducted using alternative thresholds (30%, 40%) and alternative cost indicators (direct cost only, direct medical costs only). We did not test alternative ability to pay (denominator) measures, as this would have required imputation of missing self-reported income estimates and more comprehensive asset ownership reporting.

#### Coping with TB, employment changes and social consequences

The study collected and analysed the occurrence of coping strategies to compensate for costs faced for TB care, perceived social impact and self-assessed financial impact.

#### Results reporting

In accordance with WHO reporting standards,^11^ socio-economic and model of care characteristics are presented for survey sample. Survey-adjusted results are presented for utilisation of health services, income, total costs, catastrophic cost proportions, dissaving strategies and risk factors for experiencing catastrophic costs. Descriptive statistics (mean, standard deviation [SD], median and interquartile range [IQR] and 95% confidence interval [CI]) were calculated for cost, annual household expenditure and cost drivers. However, given use of non-response weights to adjust for sampling design, median and IQR require cautionary interpretation. Proportions were calculated for sociodemographic characteristics, dissaving strategies and incidence of catastrophic costs. Costs, income and dissaving patterns were analysed across household expenditure quintile.

The odds of TB-affected households incurring catastrophic costs were evaluated against the patient’s sociodemographic and clinical characteristics using univariate and multivariate analysis. We present the unadjusted odds ratios (ORs) from univariate regressions and the adjusted odds ratios (aORs; controlling for covariates selected via a step-wise deletion regression using a drop threshold of *P* > 0.1). Statistical analyses and data visualisations were performed using Stata v15.0 (StataCorp, College Station, TX, USA) and R v4.0.1 (R Foundation for Statistical Computing, Vienna, Austria).

#### Ethics statement and details of informed consent

The study protocol was approved by the National Health Ethics Committee (*Comité National d’Éthique de la Santé*, CNES), Kinshasa, DR Congo (IOR G0008558/IRB) and the WHO African Region Ethics Review Board (AFR/ERC/2019/08.01). Written informed consent was obtained from adults and children’s guardians prior to each interview.

## RESULTS

### Study population

A total of 1,121 patients participated in the survey, of whom 1,108 were eligible (911 DS-TB, 197 DR-TB). Sampled patients included 59.6% male and 12.5% under age 15 or above 65, in line with routine surveillance patterns.^2^ Compared to 60% nationally, 65% were employed prior to contracting TB.^17^ Most patients (93.9%) had no health insurance, 7.4% received food and transport support (this was double in the case of DR-TB, 14.2%) and 49.1% were in the intensive phase; 8% were TB-HIV co-infected, while 18% did not know their status. Analysis by DR status is presented in Table 1.

**Table 1:** Sociodemographic and clinical characteristics of survey sample: first national TB patient cost survey, Democratic Republic of Congo, 2019 (n = 1108)

	DR-TB (*n*= 197)		DS-TB (*n* = 911)		Overall sample (*n* = 1,108)	
		
	*n*	(%)	*n*	(%)	*n*	(%)
Sociodemographic characteristics						
Sex						
Male	122	(61.9)	538	(59.1)	660	(59.6)
Female	75	(38.1)	373	(40.9)	448	(40.4)
Age, years						
0–14	8	(4.1)	46	(5.0)	54	(4.9)
15–24	33	(16.8)	164	(18.0)	197	(17.8)
25–34	45	(22.8)	224	(24.6)	269	(24.3)
25–44	42	(21.3)	186	(20.4)	228	(20.6)
45–54	31	(15.7)	141	(15.5)	172	(15.5)
55–64	16	(8.1)	88	(9.7)	104	(9.4)
≥ 65	22	(11.2)	62	(6.8)	84	(7.6)
Education level						
No education	30	(15)	161	(18)	191	(17)
Primary education	52	(26)	291	(32)	343	(31)
Secondary or higher	115	(58)	459	(50)	574	(52)
Occupation, pre-disease						
Formal employment	44	(23.8)	155	(18.9)	199	(19.8)
Informal employment	69	(37.3)	279	(34.1)	348	(34.7)
Unemployed	30	(16.2)	168	(20.5)	198	(19.7)
Student/housework	25	(13.5)	128	(15.6)	153	(15.3)
Self-employed	17	(9.2)	88	(10.8)	105	(10.5)
Health insurance and social assistance						
None	185	(94.4)	850	(93.8)	1,035	(93.9)
Community health insurance	6	(3.1)	31	(3.4)	37	(3.4)
Employer’s insurance	3	(1.5)	17	(1.9)	20	(1.8)
Social security for civil servants	0	—	7	(0.8)	7	(0.6)
Private insurance	2	(1.0)	1	(0.1)	3	(0.3)
Patient support (transport, food)	28	(14.2)	54	(5.9)	82	(7.4)
Household size, mean (SD)	6.9	(3.6)	6.5	(3.8)	6.6	(3.7)
Patient was main income earner prior to disease	109	(55.3)	444	(48.7)	553	(49.9)
Proportion of population living below international poverty line at $1.90/day (2011 PPP)	160	(81.2)	788	(86.5)	948	(85.6)
Clinical characteristics						
Treatment phase						
Intensive	116	(58.9)	428	(47.0)	544	(49.1)
Continuation	81	(41.1)	483	(53.0)	564	(50.9)
Recorded HIV status						
Positive	33	(16.8)	56	(6.1)	89	(8.0)
Negative	140	(71.1)	657	(72.1)	797	(71.9)
Unknown	24	(12.2)	180	(19.8)	204	(18.4)
Untested			18	(2.0)	18	(1.6)
Retreatment status						
New	34	(42.0)	462	(95.7)	496	(87.9)
Relapse	38	(46.9)	14	(2.9)	52	(9.2)
Lost to follow-up	38	(46.9)	14	(2.9)	52	(9.2)
Previously treated	5	(6.2)	5	(1.0)	10	(1.8)
TB case type						
Pulmonary TB (bacteriologically confirmed)	150	(76.1)	742	(81.4)	892	(80.5)
Pulmonary TB (clnical diagnosis)	27	(13.7)	105	(11.5)	132	(11.9)
Extrapulmonary TB	20	(10.2)	64	(7.0)	84	(7.6)

DR-TB = drug-resistant TB; DS-TB = drug-susceptible TB; SD = standard deviation; PPP = purchasing power parity.

### Utilisation of health services

Patients were interviewed at a health centre (71.5%), hospital (25.7%) or dispensary (2.8%); 5% of sampled patients were hospitalised at the time of interview and 10.9% during the treatment phase for an average of 13.3 days (95% CI 4.2–22.5) (Table 2). Hospitalisation rates and durations were higher among DR-TB patients, with 16.8% spending 15 days in hospital on average (95% CI 0–30.5) compared with 4.7% and 13 days (95% CI 5.9–19.5) for DS-TB. The period from onset of symptoms to notification ranged from 10 weeks (95% CI 8.1–12.1) for DS-TB to 11.2 weeks (95% CI 6.7–15.7) for DR-TB. Conversely, the number of medical visits prior to diagnosis was low—1.5 (95% CI 1.3–1.8) on average. Most patients (54.6%) received direct observed treatment (DOT) against 45.4% self-administering TB treatment. Over a TB episode, patients did 226 healthcare visits (95% CI 188–264) on average; however, the frequencies varied substantially by resistance status, with 350 visits (95% CI 277–423) for DR-TB compared with 152 (95% CI 138–165) for DS-TB patients.

**Table 2:** Utilisation of health services in first national TB patient cost survey, Democratic Republic of Congo, 2019 (n= 1108)

		DR-TB (*n* = 202)		DS-TB (*n* = 916)		Overall sample (*n* = 1,108)	
		
		*n*	%	*n*	%	*n*	%
Mode of TB treatment							
Self-administered		75	38.2	432	48.1	507	45.4
Directly observed treatment		122	61.8	466	51.9	610	54.6
Facility type at time of inteview							
Dispensary (*Centre de santé*)		5	2.3	26	2.9	31	2.8
Health centre (*Centre de traitement*)		140	69.1	660	72.0	799	71.5
Hospital (*Hôpital général de Référence*)		58	28.6	230	25.1	287	25.7
Hospitalisation	Hospitalised at time of interview	13	6.3	43	4.7	56	5.0
	Hospitalised during current phase	34	16.8	88	9.6	122	10.9
	Days hospitalised during current phase, mean	14.9	−0.6 to 30.5	12.7	5.9 to 19.5	13.3	4.2 to 22.5
Mean number of health facility visits	Total for one episode	564	468.4 to 659.6	151.7	138.2 to 165.1	226.2	188.2 to 264.2
	Directly observed therapy	350.1	277.0 to 423.2	85.8	73.1 to 98.4	133.6	102.2 to 165.0
	Follow-up	23.8	17.9 to 29.8	8	7.2 to 8.8	10.9	9.1 to 12.6
	Drug collection	369.3	260.0 to 478.6	113.1	97.9 to 128.4	159.9	124.5 to 195.4
	Pre-diagnosis	1.7	1.0 to 2.5	1.5	1.3 to 1.7	1.5	1.3 to 1.8
	Pre-diagnosis (non-public facility)	0.3	0.2 to 0.4	0.2	0.2 to 0.3	0.3	0.2 to 0.3
Mean treatment duration, months	Intensive phase	12	11.9 to 12.1	4	4.0 to 4.1	3.1	2.6 to 3.5
	Continuation phase	8	—	2	2.0 to 2.0	5.5	4.9 to 6.1
	Total	20	19.9 to 20.1	6.1	6.0 to 6.1	8.6	7.5 to 9.6
Mean treatment delay, weeks		11.2	(6.7 to 15.7)	10.1	8.1 to 12.1	10.4	8.5 to 12.3

DR-TB = drug-resistant TB; DS-TB = drug-susceptible TB.

### Household’s ability to pay and poverty levels prior to TB

Of all survey respondents, 50% were the main income earners prior to the disease (Table 1). Annual household expenditure, used as the denominator of the catastrophic cost indicator for DR Congo, was on average US$1,472 (95% CI 1,213–1,731; median US$1,110, IQR 566–2,012). Higher annual expenditure levels were reported among DR-TB (US$1,710, 95% CI 1,319–2,101) than among DS-TB-affected households (US$1,419, 95% CI 1,172–1,667) (Table 3).

**Table 3:** Households ability to pay (US$, 2019) and proportion of population living below US$1.90 a day, by quintile, ^*^ First National TB Patient Cost Survey, Democratic Republic of Congo, 2019 (n = 1108, survey adjusted)

	Household expenditure quintiles					

	Poorest	Second	Third	Fourth	Wealthiest	Overall
Overall						
Annual household expenditure						
Mean ± SD (95% CI)	350.3 ± 126.8 (320.9–379.6)	639.7 ± 82.4 (616.0–663.4)	1,124.9 ± 162.4 (1,090.7–1,159.2)	1,793.6 ± 278.4 (1,702.4–1,884.8)	3,469.4 ± 828.7 (3,132.6–3,806.1)	1,472.2 ± 1,186.8 (1,213.1–1,731.3)
Median [IQR]	379 [249–456]	624 [567–699]	1,122 [996–1,285]	1,747 [1,556–2,012]	3,289 [2,776–4,016]	1,110 [566–2,012]
Proportion living below the international poverty line^†^						
% (95% CI)	90.7 (85.6–94.1)	89.4 (83.5–93.3)	87.2 (80.6–91.8)	87.5 (77.5–93.5)	74.1 (61.5–83.7)	85.7 (82.6-88.4)
DS-TB						
Annual household expenditure						
Mean ± SD (95% CI)	344.8 ± 126.2 (316.8–372.8)	635.9 ± 80.3 (611.6–660.3)	1,111.9 ± 161.1 (1,059.3–1,164.5)	1,787.6 ± 270.6 (1,687.3–1,887.8)	3,440.1 ± 831.3 (3,058.4–3,821.8)	1,419.6 ± 1,171.6 (1,171.9–1,667.3)
Median [IQR]	369 [244–446]	621 [567–690]	1,107 [989–1,260]	1,746 [1,556–1,993]	3,240 [2,752–3,986]	1,029 [553–1,926]
Proportion of living below international poverty line^†^						
% (95% CI)	90.5 (85.2–94)	90.4 (84.3–94.3)	88.6 (82.5–92.8)	88.1 (77.9–94)	75.2 (57.5–87.1)	86.7 (83–89.7)
DR-TB						
Annual household expenditure						
Mean ± SD (95% CI)	393.2 ± 126.1 (290.7–495.6)	664.2 ± 92.5 (620.0–708.4)	1,163.2 ± 161.6 (1,109.0–1,217.4)	1,819.6 ± 311.7 (1,623.0–2,016.3)	3,574.5 ± 818.9 (3,177.5–3,971.5)	1,710.2 ± 1,228.3 (1,319.2–2,101.2)
Median [IQR]	444 [298–490]	685 [580–728]	1,186 [1,036–1,314]	1,749 [1,587–2,023]	3,539 [2,878–4,338]	1,318 [781–2,363]
Proportion of living below international poverty line^†^						
% (95% CI)	92.1 (59.4–98.9)	82.6 (64.6–92.5)	83 (64.2–93)	85 (62.4–95.1)	70.3 (60.8–78.4)	81.4 (68.7–89.7)

^*^ Annual household expenditure (2018 US$) quintiles are used for the distribution. However the indicator “Proportion of population living below international poverty line” is measured using self-reported income (not shown) and uses self-reported income quintiles for the distribution.

^†^ Defined at US$1.90/day (2011 PPP).

CI = confidence interval; SD = standard deviation; IQR = interquartile range; DS-TB = drug-susceptible TB ; DR = drug-resistant TB; PPP = purchasing power parity.

Pre-disease levels of poverty in 2019 were 85.7% (95% CI 83–88) in all TB-affected households, 74% (95% CI 61–84) in the wealthiest quintile, 81.4% (95% CI 69–90) in DR-TB and 86.7% (95% CI 83–90) in DS-TB patients.

### Time loss for care-seeking

On average, patients lost 428 hours in TB care; this was three-fold higher for DR-TB than for DS-TB (1,005 vs. 301), in particular in the continuation phase (Supplementary Table S1).

### Costs borne by TB-affected households: drivers and distribution across socio-economic status

The total TB episode costs incurred by affected households in 2019 averaged US$549 (95% CI 427–670), including respectively US$74 (95% CI 46–102), US$246 (95% CI 189–302) and U$229 (95% CI 155–303) for medical, non-medical and indirect costs. Patients with DR-TB incurred higher total costs (mean US$1,224, 95% CI 762–1685) than DS-TB patients (mean US$400, 95% CI 328–471). High mean episode costs among DR-TB were largely attributed to post-diagnostic travel costs (US$295, 95% CI 175–416) and food outside normal diet (US$184, 95% CI 68–300). Detailed mean and median costs are presented in Table 4 and Supplementary Table S2. TB episode costs were highest for the wealthiest, at US$819 (95% CI 424–1,213), and lowest among the second quintile (Q2) (US$451, 95% CI 267–665). The difference across quintiles was mainly attributed to non-medical costs, which more than doubled for the wealthiest (US$438, 95% CI 237–638) compared to the poorest and Q2 (US$184).

**Table 4:** TB episode costs ^*^ borne by affected households in the Democratic Republic of Congo, 2019 (2019 US$)

		DS-TB		DR-TB		Overall sample	
		
		Mean	95% CI	Mean	95% CI	Mean	95% CI
Pre-diagnosis	Medical	13.5	(11.1 to 16.0)	23	(14.3 to 31.8)	15.3	(12.3 to 18.2)
	Travel	0.8	(0.5 to 1.0)	1.5	(0.9 to 2.2)	0.9	(0.6 to 1.2)
	Accomodation	1	(0.7 to 1.4)	2.1	(0.5 to 3.7)	1.2	(0.7 to 1.7)
	Food	0.1	(0.0 to 0.2)	0.1	(−0.0 to 0.2)	0.1	(0.0 to 0.1)
	Nutritional supplements	0.1	(0.1 to 0.2)	0.1	(−0.0 to 0.2)	0.1	(0.1 to 0.2)
	Non-medical	2.1	(1.6 to 2.7)	3.5	(1.4 to 5.7)	2.4	(1.6 to 3.2)
	Hours lost by patient x hourly wage	3.5	(1.7 to 5.2)	4	(2.0 to 6.0)	3.6	(1.8 to 5.3)
Post-diagnosis	Medical	52	(23.1 to 80.1)	92	(29.7 to 153.7)	59	(31.0 to 86.7)
	Travel	73	(49.3 to 96.5)	295	(174.9 to 415.7)	113	(81.4 to 144.8)
	Accomodation	2.8	(1.4 to 4.1)	13	(−14.7 to 40.1)	4.6	(−0.8 to 9.9)
	Food	58	(32.8 to 84.1)	184	(67.8 to 300.3)	81	(48.1 to 114.2)
	Nutritional supplements	37	(23.2 to 51.2)	76	(31.3 to 121.0)	44	(27.5 to 61.1)
	Non-medical	171	(129.3 to 213.4)	568	(328.2 to 808.4)	243	(186.9 to 299.3)
	Hours lost by patient x hourly wage	157	(106.2 to 208.6)	533	(320.0 to 746.6)	225	(152.0 to 298.9)
Medical costs		65	(36.4 to 93.8)	115	(51.7 to 177.8)	74	(46.0 to 102.2)
Non-medical costs		174	(131.3 to 215.7)	572	(331.3 to 812.3)	246	(189.2 to 301.8)
Indirect costs (human capital approach)		161	(109.3 to 212.4)	537	(322.9 to 751.7)	229	(154.7 to 303.2)
Total episode costs per patient		400	(328.3 to 470.7)	1224	(762.1 to 1,685.6)	549	(427.4 to 669.8)

^*^ Indirect costs were measured using a valuation of time lost in care (human capital approach).

CI = confidence interval; DS-TB = drug-susceptible TB; DR-TB = drug-resistant TB.

### Catastrophic costs

The proportion of TB-affected households experiencing costs higher than 20% of their annual household expenditure as per the WHO global monitoring indicator^11^ was 56.5% (95% CI 49–64) for all patients and 80.2% of DR-TB affected households. The proportion varied depending on the households’ ability to pay:^*^ as poverty levels decreased, so did the proportion of households experiencing catastrophic costs, declining from the poorest to the wealthiest from 76.8% (95% CI 65–86) to 34.3% (95% CI 21–50). On sensitivity analysis, 16% (all: 95% CI 11–22), 19.3% (DR-TB: 95% CI 11–33) and 15% (DS-TB: 95% CI 11–21) of affected households experienced direct medical out-of-pocket spending (OOP) higher than 10% of their annual household expenditure in 2019 (Supplementary Table S3).

### Risk factors influencing TB-affected households incurring catastrophic costs

Households affected by drug resistance, poverty and hospitalisation had higher odds of incurring catastrophic costs than drug-susceptible, wealthiest and non-hospitalised households. The risk increased five-fold according to drug resistance status (aOR 5.10, 95% CI 3.37–7.74), ten-fold if in the poorest quintile (aOR 10.14, 95% CI 6.32–16.27) and 22 times if hospitalised (aOR 21.83, 95% CI 9.27–51.39). Other factors explored were not statistically significant (Table 5).

**Table 5:** Factors associated with TB-affected households incurring in catastrophic costs, ^*^ Democratic Republic of Congo, 2019

		Catastrophic cost incurred %	Univariate		Multivariate	
	
			Crude OR	(95% CI)	Adjusted OR	(95% CI)
Sociodemographic factors						
Age, years	0–14	65.8	Reference			
	15–24	49.8	0.52	(0.16–1.65)		
	25–34	54.8	0.63	(0.26–1.55)		
	35–44	56.5	0.68	(0.24–1.88)		
	45–54	56.7	0.68	(0.29–1.61)		
	55–64	63.2	0.90	(0.33–2.41)		
	≥65	62.5	0.87	(0.37–2.07)		
Sex	Male	55.9	Reference			
	Female	57.3	1.06	(0.76–1.46)		
Insurance	Any insurance	59.3	Reference		Reference	
	No insurance	56.3	0.88	(0.39–2.03)		
Education	Patient had no education	71.0	2.13	(1.18–3.85)		
	Patient had some education	53.5	Reference			
Employment status before TB	Employed formal	47.1	Reference			
	Employed informal	63.7	1.97	(1.06–3.64)		
	Unemployed	58.1	1.55	(0.80–3.02)		
	Retired/student/housework	54.3	1.33	(0.71–2.50)		
	Self-employed		1.74	(0.89–3.40)		
Household expenditure quintile	Poorest	76.8	6.34	(2.83–14.20)	10.14	(6.32–16.27)
	Second	65.3	3.60	(1.51–8.55)	5.45	(3.48–8.53)
	Third	61.1	3.01	(1.47–6.16)	3.72	(2.39–5.79)
	Fourth	44.3	1.53	(0.84–2.75)	1.85	(1.19–2.87)
	Wealthiest	34.3	Reference		Reference	
Patient is the main income earner Urbanicity	Yes	57.9	1.13	(0.80–1.59)	1.10	(0.83–1.45)
	Rural	63.7	Reference		Reference	
	Urban	47.4	0.88	(0.54–1.42)		
Clinical factors						
Drug resistance	DR-TB	80.2	3.86	(1.24–11.98)	5.10	(3.37–7.74)
	DS-TB	51.2	Reference		Reference	
HIV status	HIV+	63.5	Reference		Reference	
	HIV−	55.5	0.72	(0.42–1.24)	0.90	(0.53–1.54)
	HIV unknown	58.5	0.81	(0.43–1.52)	0.76	(0.42–1.40)
	HIV not tested	43.9	0.45	(0.03–7.72)	0.66	(0.19–2.28)
Hospitalisation Diagnostic delay	During the episode	95.8	21.84	(6.95–68.61)	21.83	(9.27–51.39)
	Long delay (<4 weeks before diagnosis)	63.6	1.16	(0.76–1.78)		

^*^ Defined as costs accounting for 20% or more of household expenditure.

OR = odds ratio; CI = confidence interval; DR-TB = drug-resistant TB; DS-TB = drug-susceptible TB.

### Employment changes, social consequences of TB and strategies adopted to cope

The impact of TB on employment was drastic: employment of any form among patients dropped from 65% employment to 43% while in care, with informal employment dropping from 45% to 31%, presumably due to lower licensure or sick leave protection (Figure); 23% of patients lost their jobs due to TB and 78% lost work days. Surveyed patients reported on devastating social and psychological impact, including 59% self-reporting increase in poverty levels, 48% experiencing food insecurity, 13% feeling socially excluded and 7.9% interrupting schooling; 49% (95% CI 40–58) of TB-affected households resorted to either, borrowing or selling assets to palliate the economic burden imposed by TB, with the poorest households resorting more heavily to these than the wealthiest (52.7% vs. 41.9%). Social protection reached merely 3.4% of patients. Paradoxically, the level of social protection was lowest among poor or poorest households (Table 6).

**Figure:**
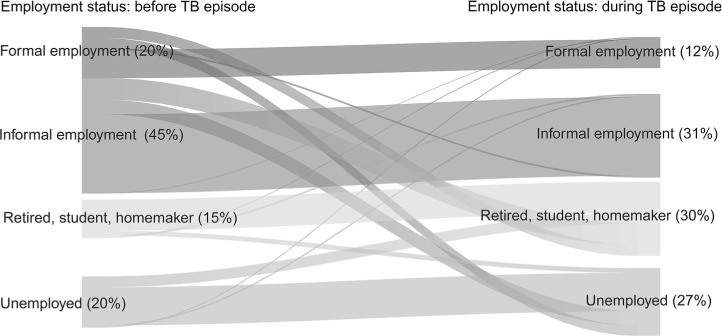
Employment changes for TB patients surveyed, First National Patient Cost Survey in Democratic Republic of Congo, 2019.

**Table 6:** Coping mechanisms and social consequences reported by participants, First National Patient Cost Survey in the Democratic Republic of Congo, 2019

	Household expenditure quintiles											

	Poorest (*n* = 226)		Second (*n* = 226)		Third (*n* = 225)		Fourth (*n* = 215)		Wealthiest (*n* = 226)		Overall sample (*n* = 1,118)	
	%	95% CI	%	95% CI	%	95% CI	%	95% CI	%	95% CI	%	95% CI
Dissaving strategy												
Loan	30.3	18.1–46.0	33.1	23.6–44.1	28.3	16.6–39.8	35.0	26.5–45.2	26.7	16.8–42.7	30.6	24.1–37.9
Sale of assets	32.2	19.9–47.3	27.5	18.7–38.4	27.2	15.2–44.0	24.5	12.7–41.1	20.8	13.7–30.2	26.4	19.7–34.4
Any of the two above	52.7	35.3–69.4	51.5	39.3–63.4	47.8	31.2–62.8	50.9	38.8–63.0	41.9	29.7–57.4	49.0	40.2–57.7
Socio-economic impact												
Food insecurity	49.9	35.8–63.2	43.9	29.9–58.7	48.6	34.5–61.1	47.3	36.5–59.6	51.1	36.9–66.3	48.2	39.3–57.1
Divorce or separated from spouse/partner	1.7	1.1–2.5	4.1	1.6–9.9	4.9	1.4–12.6	5.9	2.2–16.9	4.8	0.9–20.5	4.3	2.3–7.5
Loss of job	12.5	6.5–21.1	18.7	8.4–36.2	23.9	17.0–36.5	28.5	18.7–42.1	32.5	17.5–48.3	23.2	17.5–29.9
Child interrupted schooling	6.5	3.1–11.4	6.3	3.0–12.3	6.2	3.5–10.8	9.8	5.7–15.7	10.6	4.3–25.1	7.9	5.2–11.5
Social exclusion	10.2	5.8–17.2	12.8	5.6–26.5	18.2	10.4–28.8	12.3	5.2–25.9	12.1	5.6–25.4	13.1	8.8–19.0
Any days of work lost	80.0	71.2–85.8	76.9	57.3–89.6	81.6	67.0–88.8	78.4	67.2–88.0	74.1	61.8–84.0	78.2	70.3–84.3
Self-reported impoverishment												
Much poorer	28.0	12.6–51.1	21.0	9.0–38.4	27.3	13.7–43.8	35.0	18.4–56.1	18.5	10.3–35.9	25.6	18.3–34.4
Poorer	20.7	11.4–34.3	33.8	13.1–65.2	42.3	25.7–57.8	34.9	16.3–63.3	32.0	20.1–44.1	32.9	24.1–42.9
Unchanged	7.4	0.8–41.2	6.3	1.9–18.7	3.3	0.4–20.7	9.3	1.9–38.6	16.0	6.4–33.1	8.7	4.1–17.2
Richer	0.8	0.5–1.2	0.9	0–26.0	0		0		0		0.3	0.1–1.6
Social support after TB diagnosis												
Social protection received by household	2.8	0.4–11.4	3.1	0.4–17.6	4.5	1.7–11.1	2.4	1.0–7.3	4.4	1.5–12.2	3.4	1.7–6.7
Patient support received	4.9	1.3–17.5	6.1	2.5–13.8	5.8	1.7–17.5	6.0	2.7–12.8	14.5	6.2–30.4	7.5	4.5–12.3

CI = confidence interval.

## DISCUSSION

DR Congo relies heavily on out-of-pocket spending to finance health,^10^,^18^ and while many TB services are free, this survey highlighted that a TB episode involves OOP spending on average of US$65 for DS-TB and US$114 for DR-TB, mainly linked to hospitalisation and drug pick up frequencies. Such reliance on private spending to fund care pose serious challenges to equity of health service,^18^ and a threat to the individual and society for those unable to afford such care. Among the top WHO recommendations for new health financing compact in the COVID-19 era is the prioritisation of public funding to ensure equity of access and financial protection through a primary health care approach.

With 86% of TB patients in 2019 below global poverty levels prior to entering TB care and 56.5% of TB-affected households experiencing catastrophic costs, policies are needed geared to increase access to care and social protection among the poorest. The higher annual household expenditure among DR-TB patients and their greater access to patient support (14% vs. 6% for DS-TB) may indicate that the poorest affected by DR-TB might not be accessing care or patient support, reinforcing previous findings on utilisation of services by Laokri et al.^10^

Survey results can be compared to recent national surveys conducted in seven African countries from 2015 to 2020.^2^ The percentage of TB-affected households that experienced catastrophic costs ranged from 19% (95% CI 15–25) in Lesotho to 80% (95% CI 74–85) in Zimbabwe. The share of OOP in total costs is higher in DR Congo (14%) (Table 4) than in Nigeria (9.1%), Tanzania (7.1%), Uganda (4.9%) and Lesotho (3.9%).^2^

Several countries have successfully used this survey evidence as entry point for multisectoral actions.^19^–^23^ The devastating impact of TB on patients’ livelihood, much beyond the clinical outcomes as it impacts economic and societal development, requires a coalition with social protection actors, labour legislators and occupational safety partners to mitigate the patient’s suffering while reducing poverty, protecting patients from losing their job and psychologically supporting the patient. Multisectoral engagement is now at the heart of the WHO’s End TB Strategy,^1^ and is progressively being translated by national TB programmes and partners.^24^ In 2020, DR Congo government responded forcefully to the COVID-19 crisis with social assistance measures, including cash transfers amounting to US$50 million to 2 million beneficiaries and the State provided free water and electricity services to all households for 2 months.^25^ In 2019, one in two TB-affected households experienced food insecurity and 57% in care were out of work; however, only 7.5% accessed patient support comprising of food packages and transport vouchers.

Survey evidences TB care delivery could potentially improve by shortening care-seeking time down from 10 weeks (95% CI 8.5–12.3; Table 2) , reducing the number of health facility visits (226, 95% CI 188–264; Table 2), minimising hospitalisation requirements, shortening patient hours lost in visits for DOT (133, 95% CI 102–165; Table 2) or drug recovery through strengthening DOT at community or work level, decentralisation of care, streamlining patient journeys and diagnostic delays and patient costs.

Increased access to medical care and social support schemes could start with removal of medical costs (US$74, 95% CI 46–102; Table 4), simplified reimbursements and improved awareness on mechanisms to palliate food insecurity and other social consequences. Protecting patient employment through work legislation preventing dismissals and improving workplace services for TB patients are worth exploring. Public recommendations and TB protocols such as distancing, self-quarantine, staying at home and appropriate use of care services depend largely on employment conditions and access to adequate social insurance systems for income security.

### Survey limitations

Main survey limitation inherent from cross-sectional design survey methodology has been reported and potential solutions eluded by previous implementers of WHO methodology.^11^,^19^,^26^ We examined the distribution of income and costs across spending quintiles, observing that non-direct medical costs are highest among the wealthiest quintile.

## CONCLUSION

Despite free TB services, TB-affected households in DR Congo incur on average respectively US$400 (95% CI 328–471) and US$1,224 (95% CI 762–1,686) per episode of first-line and drug-resistant TB, while 56.5% (95% CI 49–63.7) experience catastrophic costs, posing a barrier to TB diagnosis and treatment access. Reducing OOP and designing, social and labour market measures to mitigate these costs will be needed to achieve End TB targets. The relatively higher proportion of direct medical costs in DR Congo is likely a result of the use of the national minimum wage to value time loss. DR Congo’s minimum wage is among the lowest in sub-Saharan Africa.^27^ Further study is recommended to improve the valuation of time considering the impact of informal income sources.

## Footnotes

^*^ In 2019, TB incidence was 320 (range: 207–457) per 100,000 population, 180,609 cases were notified and the case detection rate was 59%.

^*^ Ability to pay is measured in this survey based on annual household expenditure. The proportion of TB-affected households with catastrophic costs was evaluated across household expenditure quintiles.
